# Impact of the dietary antioxidant index on bone mineral density gain among mexican adults: a prospective study

**DOI:** 10.1007/s11657-025-01518-3

**Published:** 2025-03-11

**Authors:** Rogelio F. Jiménez-Ortega, Tania V. López-Pérez, Adriana Becerra‑Cervera, Diana I. Aparicio-Bautista, Nelly Patiño, Guadalupe Salas-Martínez, Jorge Salmerón, Rafael Velázquez‑Cruz, Berenice Rivera‑Paredez

**Affiliations:** 1 Laboratorio de Genómica del Metabolismo Óseo, Instituto Nacional de Medicina Genómica (INMEGEN), 14610 Mexico City, Mexico; 2 Clínica Integral Universitaria (CIU), Universidad Estatal del Valle de Ecatepec (UNEVE), Ecatepec de Morelos, 55210 Mexico, Mexico; 3 Consejo Nacional de Humanidades, Ciencias y Tecnologías, (CONAHCYT), 03940 Mexico City, Mexico; 4 Unidad de Citometría de Flujo (UCiF), Instituto Nacional de Medicina Genómica (INMEGEN), 14610 Mexico City, Mexico; 5 Laboratorio de Inmunogenómica y Enfermedades Metabólicas, Instituto Nacional de Medicina Genómica (INMEGEN), 14610 Mexico City, Mexico; 6 Centro de Investigación en Políticas, Población y Salud (CIPPS), Facultad de Medicina, Universidad Nacional Autónoma de México (UNAM), 04510 Mexico City, Mexico

**Keywords:** Antioxidants, Vitamins, Minerals, Dietary antioxidant index, Bone mineral density, Older adults

## Abstract

***Summary*:**

In the Mexican population, low dietary antioxidant intake (DAI) is associated with reduced bone mineral density (BMD). A decline in DAI over time further contributes to BMD loss, particularly at the total hip, femoral neck, and lumbar spine, with a more pronounced effect in women over 45 years old.

**Purpose:**

Bone remodeling, balancing resorption and formation, is crucial for bone health. Aging shifts this balance, reducing BMD and increasing osteoporosis risk. Reactive oxygen species (ROS) contribute to bone loss through oxidative stress. Antioxidants may help mitigate this damage, but their impact on BMD in populations with inadequate nutrient intake, like Mexicans, needs to be better understood. This study explores the association between DAI changes and BMD in a Mexican population.

**Methods:**

Data were sourced from the Health Worker Cohort Study (HWCS), including 1,318 participants (aged ≥ 20) with BMD measurements and complete dietary information at two time points. The study employed a longitudinal design was used, whit data from two waves of the study (2010–2012 and 2017–2019), providing a median follow-up time of 6.4 years for men and 6.8 years for women. Dietary antioxidant intake was assessed using a validated Food Frequency Questionnaire for the Mexican diet. BMD was measured at the femoral neck, total hip, and lumbar spine using dual-energy X-ray absorptiometry (DEXA). Fixed-effects regression models were applied to analyze the association between DAI and BMD at different sites, adjusting for time-varying covariates.

**Results:**

Changes in DAI scores were associated with lower BMD at various sites. Each unit decrease in DAI over time was associated with a BMD loss of -0.002,-0.004 g/cm^2^ at the total hip, femoral neck, and lumbar spine. Notable declines were observed in women, particularly those over 45 years old, where specific antioxidant components, like zinc, magnesium, and selenium, were linked to lower BMD.

**Conclusion:**

This study underscores the role of reduced dietary antioxidant intake in contributing lower BMD, particularly among older adults. Diets low in antioxidant may increase the risk of osteoporosis, especially in populations with insufficient nutrient intake.

**Supplementary Information:**

The online version contains supplementary material available at 10.1007/s11657-025-01518-3.

## Introduction

Bone is a dynamic organ regulated through continuous remodeling to maintain the homeostatic balance between bone resorption orchestrated by osteoclasts and bone formation orchestrated by osteoblasts [1]. Throughout life, destruction processes begin to predominate, leading to a gradual loss of bone mineral density (BMD) and causing bone fragility, which can result in the development of diseases such as osteoporosis (OP) [2]. Metabolic process at the cellular level, such the activation of mitochondrial and cytoplasmic enzymes, lead to oxidation reactions and the formation of free radicals, including ROS [3]. ROS can also produce harmful products that accumulate and cause structural damage to cells. In osteoblasts and osteocytes, an increase in ROS causes genomic DNA damage, apoptosis, and an upregulation of osteoclastogenesis and osteoclast activity [4]. Enzymes such as lipoxygenase-dependent lipid peroxidation activated by ROS play an important role in bone loss associated with aging [5]. An imbalance between ROS production and antioxidants can lead to oxidative stress (OS).

In this sense, antioxidants have emerged as potential therapeutic agents to mitigate bone damage caused by excess ROS. These compounds are known for their ability to eliminate and neutralize ROS, offering a promising approach for treating bone metabolism diseases [6]. The Dietary Antioxidant Index (DAI) is a tool designed to assess the overall antioxidant content of an individual's diet. Unlike traditional methods that focus on the intake of individual antioxidants, the DAI considers the total antioxidant capacity of the diet, taking into account not only the quantity of antioxidants consumed but also their collective ability to counteract the harmful effects of oxidative stress. This index aims to capture the combined impact of antioxidants found in foods, including vitamins, minerals, and other bioactive compounds, providing a more comprehensive picture of the antioxidant quality of the diet. A higher DAI indicates that an individual consumes a diet rich in antioxidant-containing foods, such as fruits, vegetables, nuts, and whole grains, which may be linked to better protection against oxidative stress and, improved bone health. Several studies have shown that a diet rich in antioxidants is associated with higher BMD, suggesting that the DAI could be a useful indicator of bone health [7–10]. A higher DAI indicates that an individual consumes a diet rich in antioxidant-containing foods, such as fruits, vegetables, nuts, and whole grains, which may be linked to better protection against oxidative stress and, consequently, improved bone health [11]. Several studies have shown that a diet rich in antioxidants is associated with higher BMD, suggesting that the DAI could be a useful indicator of bone health [11]. For example, Han et al*.*, 2023, mention that the DAI positively correlates with the BMD of the femoral neck, truncal, and total spine, suggesting that an intake rich in antioxidants can reduce the risk of low BMD [12]. Chen Y et al*.*, 2023, report that CDAI is inversely associated with OP in US adults aged 40 to 85 [10]. Solgi et al. 2023, suggest that adherence to a diet rich in antioxidants protects against OP in Iranian postmenopausal women, based on a case–control study [13]. A study conducted by De França et al*.* observed that a higher intake of vitamin A was associated with a reduction in BMD; however, this association disappeared when other antioxidants were considered together [14], leading to the conclusion that a single antioxidant nutrient does not represent the total antioxidant capacity of the diet. Thus, an antioxidant-based dietary approach may be beneficial for the prevention and treatment of osteoporosis, as the negative effect of vitamin A on BMD was neutralized by the intake of other antioxidant nutrients. The consumption of antioxidants is essential to maintain bone health [15]. However, food culture varies significantly between regions and countries due to various historical, geographical, climatic, cultural, and economic influences. For example, although malnutrition has decreased in Mexico, the diet has changed from a traditional diet rich in corn products, beans, fruits, and vegetables to a diet rich in energy-dense, nutrient-poor products, and empty calories. Hence, the usual intake of vitamins and minerals is likely lower than the population's needs, which could lead to the development of various diseases [16].

The Mexican National Health and Nutrition Survey (ENSANUT) has revealed that the Mexican population has nutritional deficiencies [17], especially in micronutrients. Analysis of ENSANUT data using the nutrient retention factor (NRF) showed that nutrient deficiencies among Mexicans are even higher than reported without this adjustment, particularly in adults [18]. Pedroza-Tobias et al*.* also found that women have nutrient deficiency slightly higher than men, which is relevant because a variable such as sex is also a critical factor in BMD [19]. Therefore, this study aims to explore the association of changes in DAI with changes in BMD in the Mexican population, using data from the Health Worker Cohort Study (HWCS), which includes longitudinal data from two waves: the first wave in 2010–2012 and the second wave in 2017–2019.

## Methods

### Study population

Data were sourced from the HWCS, including 1,318 participants (aged ≥ 20) with BMD measurements and complete dietary information at two-time points. The study employed a longitudinal design with baseline data collected between 2010–2012, and follow-up data collected between 2017–2019, providing a median follow-up time of 6.7 years. Dietary antioxidant intake was assessed using the Mexican diet's validated Food Frequency Questionnaire (FFQ). BMD was measured at the femoral neck, total hip, and lumbar spine using dual-energy X-ray absorptiometry (DEXA). Fixed-effects regression models were applied to analyze the association between DAI and BMD at different sites, adjusting for time-varying covariates [20].

Participants and their relatives were recruited through the Mexican Institute of Social Security (IMSS) in Cuernavaca, Morelos. Initially, 1,760 adults aged 20 or older met the eligibility criteria at Wave 1 (2004–2010). At this stage, 217 participants were excluded for various reasons, including missing data on BMD (*n* = 78), incomplete dietary information (*n* = 95), and missing data for physical activity (*n* = 3) and smoking status (*n* = 41). This resulted in 1,543 participants at the beginning of Wave 2 (2010–2012). At Wave 2, an additional 225 participants were excluded due to missing data on BMD (*n* = 135), incomplete dietary information (*n* = 88), and missing data for physical activity (*n* = 2). After applying these exclusions, the analytic sample included 1,318 participants with complete data on BMD, dietary information, physical activity, and smoking status across both waves, The exclusion rate was similar for men and women (22.7% for men and 25.7% for women) (Supplemental Fig 1).The study protocol was approved by the Research, Ethics, and Biosecurity Committee of IMSS (12CEI 09 006 14), and all participants provided written informed consent.

### Exposure: Dietary Antioxidant Index (DAI)

Dietary intake was assessed using a semiquantitative FFQ, which captured data on the frequency of consuming 116 foods over the past 12 months. Participants reported consumption frequency on a scale ranging from "never" to "6 or more times a day". This FFQ has been previously validated in individuals from Mexico City [21]. Energy and nutrient intake were estimated using a database specific to Mexican food content [22]. DAI was calculated based on the intake of six nutrients with antioxidant properties, including three vitamins (A, C, and E) and three minerals (selenium, magnesium, and zinc), as derived from the FFQ. DAI scores were energy-adjusted using the residual method [23]. This scoring system followed the approach proposed by Wright et al*.*, each dietary vitamin and mineral was standardized by subtracting the overall mean and dividing by the global standard deviation to estimate DAI. The DAI was then calculated by summing up these standardized intakes and weighing them equally [7].

### Outcome: bone mineral density

BMD measurements were conducted using a Lunar DPX NT DEXA device (Lunar Radiation Corp., Madison, WI, USA) by trained examiners. The same DEXA machine was utilized during both study periods (2004–2006 and 2010–2012). Measurement sites included the femoral neck, total hip, and lumbar spine (L1–L4). Quality control checks were performed daily by trained technicians using the manufacturer's phantom. The daily coefficient of variation met standard operational criteria, and the in vivo coefficient of variation was maintained below 1.0–1.5%.

### Other covariates

Demographic details such as age, sex, medication use (specifically, hormone replacement therapy (HRT)), calcium supplement intake, smoking habits, physical activity, and dietary habits were all gathered through self-administered questionnaires during both study periods [20]. Age at baseline was categorized as < 45 and ≥ 45 years, informed by previous research indicating hormonal changes affecting BMD around age 45 [24]. Smoking status was classified as never smokers, former smokers, and current smokers. Leisure-time physical activity (LTPA) levels were estimated using a validated PA questionnaire [25], with categories defined as inactive (< 150 min/week of moderate to vigorous activity) or active (≥ 150 min/week) [26]. Type 2 diabetes (T2D) was defined based on self-reported physician diagnosis, use of hypoglycemic medication, or fasting glucose levels exceeding established cut-off points. Height and weight were measured using standardized procedures to calculate body mass index (BMI) (kg/m^2^) at each assessment wave, with classification based on WHO BMI guidelines [27].

### Statistical analysis

Analyzing descriptive statistics, categorized by gender and wave, included computing central tendency for continuous variables and frequencies for categorical variables. Differences between waves were assessed using matched pairs T-tests for continuous variables and McNemar's test for categorical variables. The components of the DAI were energy-adjusted using the residual method [23].

Sex-specific fixed-effects regression models were employed to investigate longitudinal associations between DAI, its individual components, and BMD at different sites (total hip, femoral neck, and lumbar spine). These models evaluated DAI and its components continuously and categorically, using quintiles to analyze changes in exposure over time. The DAI categories (very low, low, medium, high, and very high) were derived from the quintiles of antioxidant intake calculated from the DAI. These categories were used to examine changes in DAI over time. The term "change in categories" refers to participants who experienced a shift in their DAI consumption category over time. For example, an individual may have been in the highest DAI consumption category at baseline (Wave 1) and, at follow-up (Wave 2), moved to the lowest category.

In the models, we focused on the decrease in DAI over time rather than an increase. Specifically, the fixed-effects models were adjusted for a decrease of 1 unit in DAI. Additionally, the highest DAI category ("very high") was used as the reference group when examining category changes. This approach allows us to assess how changes in antioxidant intake (either decreases or shifts in categories) are associated with changes in BMD, using each participant as their own control. In this way, we can examine the effects of variations in antioxidant intake on BMD more accurately. The models were adjusted for BMI, smoking status, LTPA, calcium supplements, calcium intake, vitamin D intake, T2D, and HRT. In the analysis for women, age at baseline (< 45 years vs ≥ 45 years) served as a proxy for postmenopausal status. STATA version 14.0 statistical software was utilized for all analyses [28]. All statistical tests were two-tailed, and significance was set at *p* < 0.05.

## Results

Our analytic sample comprised 1,318 participants with a median age of 46.0 years (P25-P75 37–55), primarily women (75.2%). The mean follow-up time for participants was 6.7 years (SD 0.93). Follow-up duration was similar for both men and women, with a median of 6.4 years (SD 1.2) for men and 6.8 years (SD 0.82) for women. As shown in Table 1, overweight and obesity were classified according to the World Health Organization (WHO) criteria using BMI values. The prevalence of overweight and obesity was 47.4% and 19.9% in women and 41.2% and 18.1% in men, respectively. It is important to note that BMI does not distinguish between fat and lean mass and thus may not fully reflect the risks associated with obesity. To improve the accuracy of body composition assessment, we included body fat percentage measurements obtained through DEXA, which provides more precise data. However, there are no universally accepted cut-off points for body fat percentage, making interpretation complex (Table 1).

**Table 1:** Descriptive statistics of the 1,318 participants from health workers cohort study

	Males (*n* = 327)		Females (*n* = 991)	
Characteristics	Baseline	Follow-up	Baseline	Follow-up
	Median (P25-P75)	Median (P25-P75)	Median (P25-P75)	Median (P25-P75)
Age (years)	45(36–54)	51(43–61)	46(37–55)	53(44–62)
Education, %				
Elemental	25.4	-	32.0	-
Middle school	19.3	-	22.8	-
High school or higher	52.0	-	41.6	-
BMI (kg/m^2^)	26.5(24.3–29.2)	26.9(24.4–29.5)*	25.8(23.4–28.6)	26.4(23.8–29.3)*
Nutritional status, %				
Overweight, %	47.4	47.7	41.2	42.1
Obesity, %	19.9	20.5	18.1	20.7
Body fat proportion	30.6(27.3–35.0)	32.3(28.9–35.9) *	42.9(38.5–46.9)	44.6(40.7–48.8)*
Femoral neck BMD (g/cm^2^)	1.032(0.947–1.139)	1.00(0.911–1.115)*	0.957(0.867–1.054)	0.929(0.830–1.025)*
Femoral neck T-score, SD	−0.26(−0.90,0.59)	−0.51(−1.21,0.36)*	−0.50(−1.31,0.31)	−0.81(−1.52,−0.09)*
Low BMD, %	22.3	31.2	34.3	45.4
Hip BMD (g/cm^2^)	1.089(0.997–1.183)	1.067(0.974–1.160)*	0.995(0.904–1.087)	0.958(0.868–1.057)*
Hip T-score, SD	−0.07(−0.71,0.59)	−0.22(−0.88,0.42)*	−0.11(−0.85,0.65)	−0.40(−1.12,0.40)*
Low BMD, %	17.4	21.6	20.8	28.4
Lumbar spine BMD (g/cm^2^)	1.149(1.039–1.260)	1.152(1.053–1.278)	1.107(0.996–1.216)	1.077(0.960–1.193)*
Lumbar spine T-score, SD	−0.78(−1.66,0.10)	−0.77(−1.58,0.21)	−0.75(−1.64,0.15)	−1.02(−1.93,−0.07)*
Low BMD, %	42.8	41.6	42.7	50.4
Diabetes, %	13.1	17.1	11.8	15.4*
Smoking status, %				
Current, %	22.6	16.5	13.6	10
Past, %	39.5	50.2*	22.6	28.4*
Leisure time physical activity (min/day)	46.8	41.3	35.2	32.1
Calcium supplement,%	4.6	0	17.6	18.2
Diet				
Total energy (kcal/day)	2051(1509–2694)	1863(1387–2359)*	1934(1497–2476)	1688(1244–2183)*
Alcohol (g/day)	4.0(1.0–11.4)	2.8(0.8–7.6)*	0.8(0.04–2.2)	0.8(0–1.8)*
Vitamin D intake (IU/day)	182.1(117.2–305.9)	136.5(73.7–231.6)*	204.0(135.4–325.7)	142.8(87.1–257.7)*
Calcium intake (mg/day)	917(652–1294)	756(522–1062)*	931(697–1336)	753(513–1048)*
Dietary antioxidant index (DAI)	−0.19(−1.92,1.36)	−2.37(−4.08,−0.60)*	−0.35(−1.95,1.39)	−2.66(−4.33,−0.80)*
Selenium, µg/d	53.3(42.9–66.8)	44.3(36.0–56.2)*	48.1(38.4–58.4)	38.9(29.9–48.5)*
Zinc, mg/d	9.2(8.2–10.5)	6.6(6.0–7.6)*	8.8(7.8–10.1)	6.4(5.7–7.3)*
Vitamin A, µg/d	1424(1085–1821)	1315(994–1826)	1910(1388–2573)	1697(1224–2261)*
Vitamin C, mg/d	226(155–292)	208(149–281)	288(201–392)	253(178–330)*
Vitamin E, mg/d	7.1(6.2–8.1)	6.4(5.6–7.5)*	7.3(6.3–8.4)	6.8(5.7–7.9)*
Magnesium, mg/d	365.4(327–409)	336(292–381)*	372(333–418)	325(289–369)*
Fiber intake (g/day)	24.8(18.1–34.1)	24.8(18.6–33.0)	25.9(18.8–35.3)	23.8(17.9–31.6)*
Hormone Replacement Therapy, %	-	-	6	4.8

*p* values from Paired sample t-test (continuous variables) or McNamar’s test (categorical variables). * *p* < 0.05. The mean follow-up time for participants was 6.7 years. The "Nutritional Status" refers to the classification of participants according to BMI categories as defined by the World Health Organization (WHO), BMI of 25–29.9 were categorized as overweight, and those with a BMI ≥ 30 as obese. Low BMD: a BMD T-score of less than − 1 SD. The Recommended Dietary Allowance (RDA) for Vitamin D is 600 IU, for Calcium 1000 mg, for Selenium 55 µg, for Zinc 11 mg for males and 8 mg for females, for Vitamin A 900 µg for males and 700 µg for females, for Vitamin C 90 mg for males and 75 mg for females, for Vitamin E 15 mg, and for Magnesium 420 mg for males and 320 mg for females

**Fig. 1:**
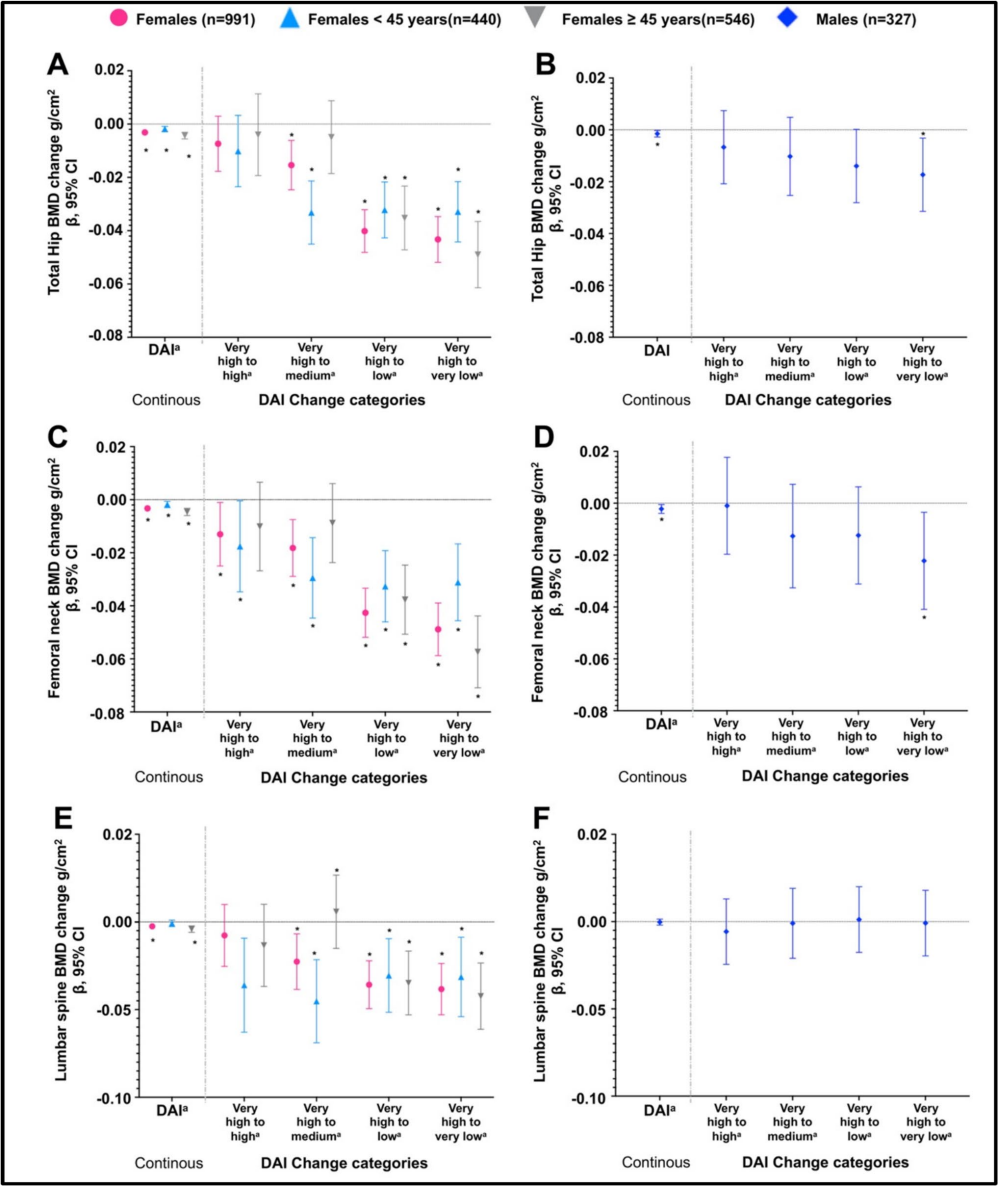
Association between DAI and different BMD sites by sex and age categories. **A**) Association between DAI and Total hip BMD among females, **B**) Association between DAI and Total hip BMD among males, **C**) Association between DAI and Femoral neck BMD among females, **D**) Association between DAI and Femoral neck BMD among males, **E**) Association between DAI and Lumbar spine BMD among females, **F**) Association between DAI and Lumbar spine BMD among males. ^a^DAI categories were defined based on quintiles, with 'very low,' 'low,' 'medium,' 'high,' and 'very high' representing different levels of antioxidant intake. "Category change" refers to participants who moved between different quintiles of DAI over time. For example, an individual may have been in the highest DAI quintile at baseline and moved to a lower quintile at follow-up. The relationship between DAI category change and bone mineral density (BMD) is assessed across different BMD measurement sites (total hip, femoral neck, and lumbar spine). Error bars represent 95% confidence intervals. *p value < 0.05

Figure 1 illustrates the intra-individual or longitudinal association between DAI and different sites of BMD by sex and age categories in women. For each decrease of one unit in the DAI score between baseline and follow-up measurements, a loss of −0.002 g/cm^2^ (95%CI: −0.003,−0.0002) was observed in men, −0.003 g/cm^2^ (95%CI, −0.004,−0.002) in women, −0.002 g/cm^2^ (95%CI, −0.003, −0.0009) in women < 45 years, and −0.004 g/cm^2^ (95%CI: −0.006,−0.003) in women ≥ 45 years in total hip BMD (Fig. 1A and B).

Similar associations were observed for the femoral neck BMD (Fig. 1C and D). However, for the lumbar spine BMD and DAI continuous, a significant association was only observed in the total women group and women ≥ 45 years (β = −0.003; 95%CI: −0.004,−0.001 and β = −0.004; 95%CI −0.006,−0.002; respectively) (Fig. 1E and F). It was also observed that individuals who reduced their DAI consumption category (defined by quintiles) over time tended to have, on average, lower BMD at different skeletal sites.

Finally, we explored the role of DAI components at different BMD sites. For all women and women over 45 years old, we observed that all components were associated with BMD loss at various sites. However, for men, only some components were statistically significant.

For instance, decreased consumption of zinc, vitamin E, selenium, and magnesium were associated with lower BMD, but not across all sites. Conversely, decreased vitamin A consumption was associated with higher BMD at the total hip in males. Among women under 45, we found that vitamin A, magnesium, zinc, vitamin E, and selenium intake were associated with lower BMD at the total hip or femoral neck. Most DAI components were significant when the variables were analyzed as continuous, likely due to the sample size (Supplementary Table 1–6).

## Discussion

To our knowledge, this is the first study to investigate the relationship between the DAI and BMD in Mexican adults. Although the coefficients observed were relatively small, it is important to recognize that even modest improvements in BMD can have significant clinical implications. Small decreases in BMD, particularly in older adults or those at risk for osteoporosis, can increase the risk of fractures and compromise bone health. The cumulative effect of dietary antioxidants over time, even if modest on a per-unit basis, may not be sufficient to counteract significant bone loss or prevent osteoporosis in the long term. Therefore, given that the decrease in DAI has a modest effect on the decline in BMD, these findings suggest that dietary antioxidants may still contribute to bone health, although the impact is relatively small. While the observed changes in BMD are modest, even small reductions in bone density over time can have clinical implications, particularly in populations at risk for osteoporosis. Since dietary changes and nutrient intake adjustments are achievable with minimal adverse effects, promoting antioxidant-rich diets could still offer a valuable strategy for supporting bone health. Thus, despite the modest effect of DAI reduction on BMD, these findings suggest that dietary antioxidants may play a role in osteoporosis prevention and management and should be considered as part of broader public health and nutritional strategies.

Our first observation was that from baseline, our population showed a reduction of hip and femoral neck BMD over time in both sexes and a reduction of lumbar spine BMD among women. This decline is consistent with the expected trajectory of BMD following peak bone mass attainment, which typically occurs around age 30 [29]. Factors such as the high prevalence of overweight and obesity in our population may exacerbate this decline. As shown in Table 1, overweight and obesity were classified according to the World Health Organization (WHO) criteria using BMI values. The prevalence of overweight and obesity was 47.4% and 19.9% in women, and 41.2% and 18.1% in men, respectively. It is important to note that BMI does not distinguish between fat mass and lean mass, and thus may not fully reflect the risks associated with obesity [30–32]. To improve the accuracy of body composition assessment, we included body fat percentage measurements obtained through DEXA, which provides more precise data. However, there are no universally accepted cut-off points for body fat percentage, making interpretation complex. Obesity promotes a pro-inflammatory environment by increasing TNF-α, IL-6, IL-1, leptin, and resistin levels that reach the bloodstream and generate a systemic inflammatory state [33]. Pro-inflammatory mediators and ROS generated by inflammation induce osteoclast activation and apoptosis in osteoblasts since ROS affect various enzymes, proteins, and cytokines involved in the coupling of osteoblasts and osteoclasts, which could lead to increased resorption activity and low bone formation characteristics of OP [34]. Recently, it has been reported that a pro-inflammatory diet enhances oxidative stress and inflammatory cytokines [35].

Furthermore, a reduction in BMD femoral areas has been observed in the American population with a high dietary inflammatory index, and this condition was associated with a higher risk of OP in the femoral neck and total femur [36]. Another explanation for the reduction in BMD could be decreased DAI intake over time. A low DAI intake is associated with reduced BMD [10, 12–14]. In contrast, an increase in DAI is positively related to total spine BMD in both men and women and has positive associations at the trochanter, femoral neck, and total femur BMD [37]. Interestingly, we observed that a decrease in DAI was associated with decrease in BMD. The reduction in BMD due to decrease DAI intake was greater in women than in men at the total hip. Additionally, postmenopausal women experienced a decline in BMD at the total hip and lumbar spine compared to premenopausal women.

Women are more susceptible to osteoporosis than men due to the influence of sexual hormones on bone homeostasis. Estrogens (E2) and androgens are crucial in bone mass development throughout life. Androgens can suppress osteoclastogenesis [38], while the E2 prevents bone resorption, inducing apoptosis in osteoclasts via the Fas ligand and raising osteoblast activity. Additionally, it has been suggested that E2 protects from oxidative stress by induction of antioxidant enzyme expression [39]. After menopause, E2 levels abruptly decline, but in older men, their E2 concentration is enough to maintain bone homeostasis [40]. Since the natural loss of E2 in women makes them more susceptible to developing OP, the fact that Mexican women additionally have dietary deficiencies [19] that promote BMD loss requires attention to address this critical health issue.

It is suggested that in our population, women have the most significant impact on BMD at several sites when they decrease their intake of antioxidants, especially postmenopausal women. The greater the decrease in antioxidant intake, as measured by the DAI, the more significant the decrease in BMD. In the Korean female population, dietary total antioxidant capacity (TAC) has been reported to protect against the OP risk in postmenopausal women but not in premenopausal women. Moreover, TAC was positively associated with BMD in the total femur, lumbar spine, and femoral neck in postmenopausal women; however, in premenopausal women, only in the lumbar spine and total femur [41].

The mechanisms through which the DAI influences BMD focus on the ability of antioxidants to mitigate oxidative stress and inflammation, factors that negatively impact bone health. Antioxidants, such as vitamins C and E, along with minerals like zinc and selenium, act by neutralizing free radicals and reducing the production of pro-inflammatory cytokines, such as TNF-α and IL-6, which promote osteoclast activity and apoptosis of osteoblasts [42]. Additionally, some antioxidants stimulate the differentiation of osteoblasts and the synthesis of bone matrix through the regulation of key genes involved in bone formation, such as *RUNX2* and *BMP2* [42]. Thus, a higher intake of antioxidants, reflected in an elevated DAI, is associated with improved BMD, especially in vulnerable populations like postmenopausal women, who experience accelerated bone mass loss due to declining estrogen levels and increased oxidative stress [43].

In our study, all components of the DAI in women, particularly postmenopausal women, were associated with a decrease in BMD at several sites. However, only some DAI components were significantly associated with BMD in men. It is important to note that we observed a low intake of vitamins A and C in men, in concordance with other studies where vitamin A intake is linked to higher BMD at the total hip [37, 44]. De França et al*.* analysed vitamin A separately in a cross-sectional study and found that a higher vitamin A intake was associated with a reduction in BMD in postmenopausal women [14]. In contrast, Rivas et al. conducted a cross-sectional study and found no association between vitamin A intake and BMD [44]. The inconsistent associations between vitamin A and BMD may be attributed to differences in study design and dietary assessment methods.

Studies have shown that dietary vitamin C intake is related to higher BMD of the lumbar and femoral neck [45], but our results in women showed an inverse relationship, with lower vitamin C intake associated with a decrease in BMD. Similarly, our findings indicate a negative relationship between vitamin E intake and BMD in the hip, femoral neck, and lumbar spine of women, but not in men. This contrasts with previous studies that found higher serum vitamin E levels to be linked to greater BMD [46], particularly in the total femur and lumbar spine [16]. The interactive effect of vitamin E with BMD may be influenced by factors such as age, gender, ethnicity, and sex [47].

Finally, we found that higher intakes of zinc, selenium, and magnesium were associated with lower BMD, but not at all sites. High intakes of zinc and selenium have been associated with higher BMD in the total femur, trochanter, and intertrochanter, as well as a lower risk of OP [37, 48]. High dietary selenium intake is associated with increased BMD at the femur, femoral neck, trochanter, intertrochanter, and lumbar spine. In addition, an inverted U-shaped relationship was observed between dietary selenium intake and BMD [49]. The positive association between Zn intake and the BMD values of the total hip and femoral neck in both men and women and of the lumbar spine only in women may be because men aged 18 to 40 years have a higher density of vertebrae in the neck and lumbar spine than women [50]. Therefore, although zinc influences the BMD in men's lumbar and cervical spines, this is not as evident as in women. We observed a negative association between magnesium intake and total hip and lumbar spine BMD levels in women over 45; however, no association was observed in the male group. We hypothesize that biological differences between the sexes may explain why only women experienced a decline in BMD with lower Mg intake. Mg is potentially essential for the metabolism of sex hormones during aging, such as testosterone, progesterone, and insulin-like growth factor 1 [51], where some studies have shown that only in men does Mg act on muscle through the endocrine system. Since these hormones are sex-dependent, the effects of Mg intake on bone may differ between sexes [52].

Some studies have reported a positive association between Se, BMD, and osteoporosis risk [53, 54]. Our study observed that low Se consumption shows a negative association with BMD levels at the total hip, femoral neck, and lumbar spine in the female population, particularly in women over 45 years of age, but not in men. E2 is involved in the regulation of osteogenesis, and it is possible that in postmenopausal women, low selenium intake is associated with low levels of BMD and, therefore, with the development of diseases such as osteoporosis. High selenium intake is associated with high levels of BMD, as reported in the literature [53, 54] and is consistent with our findings. Therefore, the effects of selenium intake on bone may also differ between sexes.

The present study has several strengths. This is the first longitudinal study to assess the relationship between DAI intake and BMD in the Mexican population. One of our key strengths lies in our rigorous methodology, including a robust fixed-effects regression model. This model is particularly advantageous as it allows us to effectively control for inherent individual-level factors that remain constant over time, such as genetic predispositions or unmeasured lifestyle habits. By doing so, we enhance the precision of our estimates and minimize potential biases that could arise from these factors. We used the DAI to assess dietary interactions beyond individual components thoroughly. Additionally, we analyzed specific antioxidant components to correlate our findings with existing literature. Our study also has some limitations. Dietary intake data may be prone to non-differential measurement errors despite using a validated FFQ designed specifically for the Mexican population. Moreover, our analysis adjusted for multiple confounding factors, although residual confounding remains a potential issue. Our study explores associations but cannot establish causal relationships. As an observational study, it aims to identify potential links between DAI and BMD rather than determine cause-and-effect. Our study lacked national representativeness and may reflect the urban population of central Mexico. However, since our focus on establishing causal relationships, concerns about national representativeness are mitigated. Additionally, we acknowledge the failure to collect information on socioeconomic and marital status. Such factors can have a significant impact on the quality of an affordable diet, which in turn could influence bone health. It's important to note that our study needed more power to explore changes in low BMD categories (osteopenia and osteoporosis) due to minimal changes over time within these categories. Additionally, we acknowledge limited statistical power in male participants. Our study includes only data on calcium supplementation. We do not have information on other supplements, such as vitamin C or vitamin A, that participants may have consumed. This limitation is important because the intake of such supplements could potentially influence bone health and BMD outcomes. Therefore, the absence of this information may have led to an underestimation or overestimation of the association between dietary antioxidants and BMD, particularly if participants' supplement use was substantial.

## Conclusion

In analyzing the individual effect of different components of DAI on BMD, there have been inconsistencies in the findings. However, all the studies, including ours, agree that lower intake of antioxidants is associated with a decrease in BMD. This suggests that the individual effect of a specific antioxidant may not fully represent the combined effect of all antioxidants consumed in the diet. Our study found a strong association between DAI and BMD in the population we studied. The results imply that decreasing antioxidant intake from the diet may be negative effects on bone health, especially for postmenopausal women. who are at a higher risk of BMD loss.

## Supplementary Information

Below is the link to the electronic supplementary material.

Supplementary file1 (DOCX 63 KB)

## Data Availability

The datasets used and analyzed during the study are available from the corresponding author on reasonable request.
