# Supplementary material for: Impact of the dietary antioxidant index on bone mineral density gain among mexican adults: a prospective study
Source: Arch Osteoporos. 2025 Mar 11;20(1):38. doi: 10.1007/s11657-025-01518-3 (PMC11897103; doi:10.1007/s11657-025-01518-3)
Supplement: Supplementary file 1 — Supplementary file1 (DOCX 63 KB) [file 11657_2025_1518_MOESM1_ESM.docx]

**Impact of the Dietary Antioxidant Index on Bone Mineral Density gain among Mexican Adults: A prospective study.**

Rogelio F. Jimenez-Ortega^1,2^**^†^**, Tania V. Lopez-Perez^1,3^**^†^**, Adriana Becerra‑Cervera^1,3^, Diana I. Aparicio-Bautista^1^, Nelly Patiño^4^, Guadalupe Salas-Martínez^5^, Jorge Salmerón^6^, Rafael Velázquez‑Cruz^1, *^, and Berenice Rivera‑Paredez^6, *^.

^1^Laboratorio de Genómica del Metabolismo Óseo, Instituto Nacional de Medicina Genómica (INMEGEN), 14610 Mexico City, Mexico.

^2^Unidad de Acupuntura Rehabilitatoria. Universidad Estatal del Valle de Ecatepec (UNEVE), 55210 Ecatepec de Morelos, México. Mexico.

^3^Consejo Nacional de Humanidades, Ciencias y Tecnologías, (CONAHCYT), 03940 Mexico City, Mexico.

^4^Unidad de Citometría de Flujo (UCiF), Instituto Nacional de Medicina Genómica (INMEGEN), 14610 Mexico City, Mexico.

^5^Laboratorio de Inmunogenómica y Enfermedades Complejas, Instituto Nacional de Medicina Genómica (INMEGEN), 14389 Mexico City, Mexico.

^6^Centro de Investigación en Políticas, Población y Salud (CIPPS), Facultad de Medicina,Universidad Nacional Autónoma de México (UNAM), 04510 Mexico City, Mexico.

**^†^ These authors contributed equally to this work.**

**Corresponding author:**

*Berenice Rivera-Paredez.

e-mail: bereriveraparedez7@gmail.com; Phone: + 52 (55) 5622 6666, Ext. 82355.

*Rafael Velázquez-Cruz.

e-mail: rvelazquez@inmegen.gob.mx; Phone: +52 (55) 5350-1900, Fax: +52 (55) 5350-1999.

| **Supplementary Table 1.** Association between Vitamin C intake and BMD. | | | | | | | | | | | | | | | | | | | | | | |  |  |  |
| --- | --- | --- | --- | --- | --- | --- | --- | --- | --- | --- | --- | --- | --- | --- | --- | --- | --- | --- | --- | --- | --- | --- | --- | --- | --- |
|  | **Males (n=327)** | | | | | | **Females (n=991)** | | | | | **Females <45 (n=440)** | | | | | **Females >=45 (n=551)** | | | | | |  |  |  |
|  | **β (% 95 CI)** | | | | | | **β (% 95 CI)** | | | | | **β (% 95 CI)** | | | | | **β (% 95 CI)** | | | | | |  |  |  |
|  | **Total hip g/cm^2^** | | | | | | | | | | | | | | | | | | | | | |  |  |  |
| VitC | -0.00001  (-0.00004, 0.00002) | | | | | | -0.00003  (-0.00005,-0.00002)* | | | | | -0.00002  (-0.00003,-0.000001) | | | | | -0.00005  (-0.00007,-0.00002)* | | | | | |  |  |  |
| Change very high to high | 0.010  (-0.013,0.033) | | | | | | -0.017  (-0.026,-0.007)* | | | | | 0.020  (-0.0005,0.040) | | | | | 0.007  (-0.013,0.028) | | | | | |  |  |  |
| Change from very high to medium | 0.022  (-0.002,0.047) | | | | | | 0.012  (-0.002,0.027) | | | | | 0.023  (-0.0001,0.045) | | | | | 0.027  (0.004,0.050)* | | | | | |  |  |  |
| Change from very high to low | -0.021  (-0.030,0.025) | | | | | | 0.015  (0.0005,0.030)* | | | | | 0.008  (-0.014,0.031) | | | | | 0.029  (0.006,0.051)* | | | | | |  |  |  |
| Change from very high to very low | 0.001  (-0.023,0.025) | | | | | | -0.015  (-0.026,-0.003)* | | | | | -0.003  (-0.019,0.013) | | | | | -0.016  (-0.034,0.003) | | | | | |  |  |  |
|  | **Femoral neck g/cm^2^** | | | | | | | | | | | | | | | | | | | | | |  |  |  |
| VitC | -0.000004  (-0.00004,0.00003) | | | | | | -0.00003  (-0.00005,-0.00002)* | | | | | -0.00001  (-0.00003,0.00001) | | | | | -0.00005  (-0.00008,-0.00003)* | | | | | |  |  |  |
| Change very high to high | 0.021  (-0.010,0.052) | | | | | | -0.017  (-0.029,-0.005)* | | | | | 0.024  (-0.001,0.048) | | | | | 0.005  (-0.018,0.029) | | | | | |  |  |  |
| Change from very high to medium | 0.034  (0.0004,0.067)* | | | | | | 0.013  (-0.044,0.030) | | | | | 0.039  (0.011,0.067)* | | | | | 0.021  (-0.006,0.048) | | | | | |  |  |  |
| Change from very high to low | 0.012  (-0.025,0.049) | | | | | | 0.014  (-0.004,0.031) | | | | | 0.012  (-0.016,0.040) | | | | | 0.028  (0.002,0.054)* | | | | | |  |  |  |
| Change from very high to very low | -0.002  (-0.035,0.030) | | | | | | -0.013  (-0.027,0.0007) | | | | | 0.017  (-0.003,0.036) | | | | | -0.021  (-0.042,-0.0001)* | | | | | |  |  |  |
|  | **Lumbar spine, g/cm^2^** | | | | | | | | | | | | | | | | | | | | | |  |  |  |
| VitC | -0.00002  (-0.00006, 0.00002) | | | | | | -0.00003  (-0.00005,-0.000004)* | | | | | -0.00001  (-0.00005,0.00002) | | | | | -0.00004  (-0.00007,-0.000004)* | | | | | |  |  |  |
| Change very high to high | -0.031  (-0.061,-0.002)* | | | | | | -0.015  (-0.030,-0.0003)* | | | | | 0.039  (0.010,0.069)* | | | | | 0.007  (-0.024,0.038) | | | | | |  |  |  |
| Change from very high to medium | 0.0004  (-0.032,0.032) | | | | | | 0.011  (-0.010,0.033) | | | | | 0.027  (-0.006,0.061) | | | | | 0.025  (-0.010,0.061) | | | | | |  |  |  |
| Change from very high to low | -0.032  (-0.067,0.004) | | | | | | -0.001  (-0.023,0.020) | | | | | 0.037  (0.004,0.071)* | | | | | -0.020  (-0.054,0.015) | | | | | |  |  |  |
| Change from very high to very low | -0.020  (-0.051,0.011) | | | | | | -0.015  (-0.032,0.002) | | | | | 0.011  (-0.013,0.034) | | | | | -0.021  (-0.048,0.007) | | | | | |  |  |  |
| Models adjusted for BMI, leisure-time physical activity (LTPA), diabetes, smoking status, calcium supplements, vitamin D intake, calcium intake, and THR. Nutrient adjusted for energy using the residual method. **p*-value <0.05 | | | | | | | | | | | | | | | | | | | | | | |  |  |  |
| **Supplementary Table 2.** Association between Vitamin E intake and BMD. | | | | | | | | | | | | | | | | | | | | | | | | | |
|  | | | | | **Males (n=327)** | | | | | | **Females (n=991)** | | | | | **Females <45 (n=440)** | | | | | | **Females >=45 (n=551)** | | | |
|  | | | | | **β (% 95 CI)** | | | | | | **β (% 95 CI)** | | | | | **β (% 95 CI)** | | | | | | **β (% 95 CI)** | | | |
|  | | | | | **Total hip g/cm^2^** | | | | | | | | | | | | | | | | | | | | |
| VitE | | | | | -0.002  (-0.004,-0.0003)* | | | | | | -0.002  (-0.004,-0.001)* | | | | | -0.001  (-0.002,0.0004) | | | | | | -0.004  (-0.006,-0.002)* | | | |
| Change very high to high | | | | | -0.002  (-0.023,0.020) | | | | | | 0.008  (-0.003,0.020) | | | | | 0.012  (-0.006,0.031) | | | | | | 0.004  (-0.012,0.020) | | | |
| Change from very high to medium | | | | | 0.013  (-0.007,0.034) | | | | | | 0.028  (0.015,0.042)* | | | | | 0.020  (0.001,0.039)* | | | | | | 0.028  (0.009,0.047)* | | | |
| Change from very high to low | | | | | -0.009  (-0.031,0.013) | | | | | | 0.021  (0.007,0.034)* | | | | | 0.016  (-0.003,0.034) | | | | | | 0.026  (0.006,0.045)* | | | |
| Change from very high to very low | | | | | -0.011  (-0.027,0.005) | | | | | | -0.016  (-0.025,-0.006)* | | | | | -0.003  (-0.017,0.011) | | | | | | -0.028  (-0.043,-0.013)* | | | |
|  | | | | | **Femoral neck g/cm^2^** | | | | | | | | | | | | | | | | | | | | |
| VitE | | | | | -0.003  (-0.006,-0.00007)* | | | | | | -0.003  (-0.004,-0.002)* | | | | | -0.002  (-0.003,-0.00001)* | | | | | | -0.004  (-0.006,-0.002)* | | | |
| Change very high to high | | | | | 0.013  (-0.014,0.041) | | | | | | 0.007  (-0.006,0.021) | | | | | -0.0002  (-0.022,0.021) | | | | | | 0.006  (-0.013,0.024) | | | |
| Change from very high to medium | | | | | 0.025  (-0.0008,0.051) | | | | | | 0.028  (0.012,0.044)* | | | | | 0.015  (-0.008,0.038) | | | | | | 0.035  (0.014,0.057)* | | | |
| Change from very high to low | | | | | -0.0004  (-0.028,0.028) | | | | | | 0.018  (0.003,0.034)* | | | | | 0.0007  (-0.022,0.023) | | | | | | 0.032  (0.009,0.054b5b5)* | | | |
| Change from very high to very low | | | | | -0.011  (-0.031,0.010) | | | | | | -0.016  (-0.027,-0.004)* | | | | | -0.008  (-0.024,0.008) | | | | | | -0.025  (-0.042,-0.008)* | | | |
|  | | | | | **Lumbar spine, g/cm^2^** | | | | | | | | | | | | | | | | | | | | |
| VitE | | | | | -0.0009  (-0.004,0.002) | | | | | | -0.003  (-0.004,-0.0007)* | | | | | -0.000002  (-0.003,0.003) | | | | | | -0.005  (-0.008,-0.003)* | | | |
| Change very high to high | | | | | -0.013  (-0.041,0.015) | | | | | | 0.007  (-0.010,0.024) | | | | | 0.024  (-0.003,0.051) | | | | | | -0.007  (-0.031,0.017) | | | |
| Change from very high to medium | | | | | -0.025  (-0.051,0.002) | | | | | | 0.023  (0.003,0.043)* | | | | | 0.038  (0.010,0.066)* | | | | | | 0.001  (-0.026,0.029) | | | |
| Change from very high to low | | | | | -0.004  (-0.032,0.025) | | | | | | 0.018  (-0.002,0.038) | | | | | 0.028  (-0.0002,0.0055) | | | | | | 0.006  (-0.023,0.035) | | | |
| Change from very high to very low | | | | | -0.013  (-0.034,0.008) | | | | | | -0.013  (-0.028,0.001) | | | | | 0.011  (-0.009,0.031) | | | | | | -0.039  (-0.061,-0.018)* | | | |
| Models adjusted for BMI, leisure-time physical activity (LTPA), diabetes, smoking status, calcium supplements, vitamin D intake, calcium intake, and THR. Nutrient adjusted for energy using the residual method. **p*-value <0.05 | | | | | | | | | | | | | | | | | | | | | | | | | |
| **Supplementary Table 3.** Association between Vitamin A intake and BMD. | | | | | | | | | | | | | | | | | | | | | | | | |  |
|  | | **Males (n=327)** | | | | **Females (n=991)** | | | | | | **Females <45 (n=440)** | | | | | | **Females >=45 (n=551)** | | | | | | |  |
|  | | **β (% 95 CI)** | | | | **β (% 95 CI)** | | | | | | **β (% 95 CI)** | | | | | | **β (% 95 CI)** | | | | | | |  |
|  | | **Total hip g/cm^2^** | | | | | | | | | | | | | | | | | | | | | | |  |
| VitA | | 0.000001  (-0.000004,0.000006) | | | | -0.000004  (-0.000007,-0.000002)* | | | | | | -0.000004  (-0.000007,-0.000001)* | | | | | | -0.000005  (-0.000009,-0.0000006)* | | | | | | |  |
| Change very high to high | | 0.031  (0.009,0.052)* | | | | -0.005  (-0.016,0.006) | | | | | | 0.017  (-0.001,0.036) | | | | | | 0.004  (-0.014,0.022) | | | | | | |  |
| Change from very high to medium | | 0.035  (0.014,0.057)* | | | | 0.012  (-0.0006,0.025) | | | | | | 0.023  (-0.0006,0.046) | | | | | | 0.034  (0.012,0.055)* | | | | | | |  |
| Change from very high to low | | 0.016  (-0.004,0.037) | | | | 0.013  (-0.001,0.028) | | | | | | 0.006  (-0.017,0.029) | | | | | | 0.034  (0.012,0.055)* | | | | | | |  |
| Change from very high to very low | | 0.021  (0.003,0.039)* | | | | -0.015  (-0.027,-0.003)* | | | | | | 0.000002  (-0.016,0.016) | | | | | | -0.018  (-0.036,-0.0009)* | | | | | | |  |
|  | | **Femoral neck g/cm^2^** | | | | | | | | | | | | | | | | | | | | | | |  |
| VitA | | 0.000003  (-0.000003,0.000009) | | | | -0.000006  (-0.000009,0.000003)* | | | | | | -0.000004  (-0.000007,-0.000006)* | | | | | | -0.000007  (-0.00001,-0.000002)* | | | | | | |  |
| Change very high to high | | 0.037  (0.009,0.066)* | | | | -0.007  (-0.019,0.005) | | | | | | 0.012  (-0.012,0.036) | | | | | | 0.002  (-0.018,0.021) | | | | | | |  |
| Change from very high to medium | | 0.042  (0.014,0.070)* | | | | 0.013  (-0.002,0.027) | | | | | | 0.009  (-0.0214,0.038) | | | | | | 0.028  (0.004,0.051)* | | | | | | |  |
| Change from very high to low | | 0.037  (0.009,0.064)* | | | | 0.009  (-0.008,0.026) | | | | | | -0.004  (-0.034,0.025) | | | | | | 0.035  (0.012,0.058)* | | | | | | |  |
| Change from very high to very low | | 0.017  (-0.007,0.041) | | | | -0.015  (-0.029,-0.002)* | | | | | | 0.0006  (-0.020,0.021) | | | | | | -0.023  (-0.042,-0.005)* | | | | | | |  |
|  | | **Lumbar spine, g/cm^2^** | | | | | | | | | | | | | | | | | | | | | | |  |
| VitA | | -0.000003  (-0.000009,0.000003) | | | | -0.000003  (-0.000007,0.000002) | | | | | | -0.000003  (-0.000008,0.000003) | | | | | | -0.000002  (-0.000007,0.000004) | | | | | | |  |
| Change very high to high | | -0.008  (-0.037,0.020) | | | | -0.008  (-0.024,0.007) | | | | | | 0.038  (0.009,0.067)* | | | | | | -0.002  (-0.027,0.022) | | | | | | |  |
| Change from very high to medium | | -0.010  (-0.038,0.018) | | | | 0.011  (-0.007,0.029) | | | | | | 0.027  (-0.009,0.062) | | | | | | 0.027  (-0.002,0.057) | | | | | | |  |
| Change from very high to low | | -0.032  (-0.059,-0.004)* | | | | 0.009  (-0.012,0.030) | | | | | | 0.016  (-0.019,0.051) | | | | | | 0.021  (-0.008,0.050) | | | | | | |  |
| Change from very high to very low | | -0.005  (-0.029,0.019) | | | | -0.014  (-0.032,0.003) | | | | | | 0.013  (-0.012,0.038) | | | | | | -0.024  (-0.047,-0.00003)* | | | | | | |  |
| Models adjusted for BMI, leisure-time physical activity (LTPA), diabetes, smoking status, calcium supplements, vitamin D intake, calcium intake, and THR. Nutrient adjusted for energy using the residual method. **p*-value <0.05 | | | | | | | | | | | | | | | | | | | | | | | | |  |
| **Supplementary Table 4.** Association between Zinc intake and BMD. | | | | | | | | | | | | | | | | | | | | | | | |  |  |
|  | | | | | **Males (n=327)** | | | | **Females (n=991)** | | | | **Females <45 (n=440)** | | | | | | **Females >=45 (n=551)** | | | | |  |  |
|  | | | | | **β (% 95 CI)** | | | | **β (% 95 CI)** | | | | **β (% 95 CI)** | | | | | | **β (% 95 CI)** | | | | |  |  |
|  | | | | | **Total hip g/cm^2^** | | | | | | | | | | | | | | | | | | |  |  |
| Zn | | | | | -0.003  (-0.005,-0.002)* | | | | -0.006  (-0.007,-0.004)* | | | | -0.003  (-0.005,-0.002)* | | | | | | -0.008  (-0.010,-0.006)* | | | | |  |  |
| Change very high to high | | | | | -0.001  (-0.019,0.017) | | | | 0.007  (-0.003,0.018) | | | | -0.004  (-0.020,0.012) | | | | | | 0.013  (-0.001,0.028) | | | | |  |  |
| Change from very high to medium | | | | | 0.010  (-0.007,0.027) | | | | 0.010  (-0.0009,0.021) | | | | -0.009  (-0.025,0.007) | | | | | | 0.024  (0.010,0.039)* | | | | |  |  |
| Change from very high to low | | | | | 0.010  (-0.008,0.027) | | | | -0.003  (-0.014,0.007) | | | | -0.014  (-0.030,0.0034) | | | | | | 0.018  (0.003,0.033)* | | | | |  |  |
| Change from very high to very low | | | | | -0.010  (-0.024,0.004) | | | | -0.028  (-0.037,-0.019)* | | | | -0.020  (-0.034,-0.007)* | | | | | | -0.033  (-0.045,-0.021)* | | | | |  |  |
|  | | | | | **Femoral neck g/cm^2^** | | | | | | | | | | | | | | | | | | |  |  |
| Zn | | | | | -0.006  (-0.008,-0.004)* | | | | -0.006  (-0.007,-0.004)* | | | | -0.003  (-0.005,-0.001)* | | | | | | -0.008  (-0.010,-0.006)* | | | | |  |  |
| Change very high to high | | | | | 0.005  (-0.019,0.028) | | | | 0.002  (-0.010,0.015) | | | | -0.010  (-0.030,0.010) | | | | | | 0.016  (0.00004,0.032)* | | | | |  |  |
| Change from very high to medium | | | | | 0.013  (-0.010,0.037) | | | | 0.012  (-0.0001,0.025) | | | | -0.003  (-0.023,0.016) | | | | | | 0.028  (0.012,0.044)* | | | | |  |  |
| Change from very high to low | | | | | 0.009  (-0.015,0.032) | | | | -0.009  (-0.022,0.003) | | | | -0.023  (-0.043,-0.003)* | | | | | | 0.015  (-0.001,0.032) | | | | |  |  |
| Change from very high to very low | | | | | -0.020  (-0.039,-0.001)* | | | | -0.031  (-0.042,-0.02)* | | | | -0.019  -(0.036,-0.002)* | | | | | | -0.035  (-0.049,-0.022)* | | | | |  |  |
|  | | | | | **Lumbar spine, g/cm^2^** | | | | | | | | | | | | | | | | | | |  |  |
| Zn | | | | | 0.001  (-0.001,0.004) | | | | -0.004  (-0.006,-0.002)* | | | | -0.0005  (-0.003,0.002) | | | | | | -0.007  (-0.010,-0.005)* | | | | |  |  |
| Change very high to high | | | | | 0.003  (-0.023,0.029) | | | | 0.002  (-0.017,0.021) | | | | 0.009  (-0.016,0.034) | | | | | | 0.004  (-0.020,0.028) | | | | |  |  |
| Change from very high to medium | | | | | 0.017  (-0.009,0.042) | | | | 0.012  (-0.007,0.030) | | | | -0.011  (-0.026,0.023) | | | | | | 0.026  (0.001,0.050)* | | | | |  |  |
| Change from very high to low | | | | | -0.004  (-0.030,0.021) | | | | -0.002  (-0.020,0.017) | | | | -0.012  (-0.037,0.013) | | | | | | 0.002  (-0.024,0.026) | | | | |  |  |
| Change from very high to very low | | | | | 0.016  (-0.005,0.037) | | | | -0.024  (-0.041,-0.008)* | | | | -0.013  (-0.033,0.008) | | | | | | -0.034  (-0.055,-0.014)* | | | | |  |  |
| Models adjusted for BMI, leisure-time physical activity (LTPA), diabetes, smoking status, calcium supplements, vitamin D intake, calcium intake, and THR. Nutrient adjusted for energy using the residual method. **p*-value <0.05. | | | | | | | | | | | | | | | | | | | | | | | |  |  |
| **Supplementary Table 5.** Association between magnesium intake and BMD. | | | | | | | | | | | | | | | | | | | | | | | |  |  |
|  | | | **Males (n=327)** | | | | | **Females (n=991)** | | | | | | | **Females <45 (n=440)** | | | | | **Females >=45 (n=551)** | | | |  |  |
|  | | | **β (% 95 CI)** | | | | | **β (% 95 CI)** | | | | | | | **β (% 95 CI)** | | | | | **β (% 95 CI)** | | | |  |  |
|  | | | **Total hip g/cm^2^** | | | | | | | | | | | | | | | | | | | | |  |  |
| Mg | | | -0.00001  (-0.00008,0.00006) | | | | | -0.0001  (0.00015,0.00007)* | | | | | | | -0.00007  (-0.0001,-0.00002)* | | | | | -0.0001  (-0.0002,-0.00009)* | | | |  |  |
| Change very high to high | | | 0.019  (-0.003,0.040) | | | | | 0.011  (-0.002,0.024) | | | | | | | 0.007  (-0.011,0.023) | | | | | 0.002  (-0.015,0.019) | | | |  |  |
| Change from very high to medium | | | 0.029  (0.007,0.052)* | | | | | 0.016  (0.002,0.031)* | | | | | | | 0.004  (-0.015,0.023) | | | | | 0.016  (-0.004,0.036) | | | |  |  |
| Change from very high to low | | | 0.029  (0.006,0.051)* | | | | | 0.008  (-0.005,0.021) | | | | | | | -0.003  (-0.021,0.014) | | | | | 0.013  (-0.006,0.032) | | | |  |  |
| Change from very high to very low | | | 0.018  (-0.001,0.037) | | | | | -0.014  (-0.026,-0.003)* | | | | | | | -0.007  (-0.021,0.008) | | | | | -0.029  (-0.045,-0.012)* | | | |  |  |
|  | | | **Femoral neck g/cm^2^** | | | | | | | | | | | | | | | | | | | | |  |  |
| Mg | | | -0.00005  (-0.0001,0.00005) | | | | | -0.0001  (-0.0002,-0.00007)* | | | | | | | -0.00007  (-0.0001,-0.00001)* | | | | | -0.0001  (-0.0002,-0.00007)* | | | |  |  |
| Change very high to high | | | 0.031  (0.002,0.060)* | | | | | 0.007  (-0.008,0.022) | | | | | | | 0.005  (-0.017,0.028) | | | | | -0.002  (-0.020,0.017) | | | |  |  |
| Change from very high to medium | | | 0.035  (0.005,0.066)* | | | | | 0.018  (0.0007,0.035)* | | | | | | | 0.003  (-0.020,0.026) | | | | | 0.024  (0.002,0.046)* | | | |  |  |
| Change from very high to low | | | 0.026  (-0.005,0.057) | | | | | 0.004  (-0.012,0.019) | | | | | | | -0.013  (-0.035,0.008) | | | | | 0.018  (-0.003,0.039) | | | |  |  |
| Change from very high to very low | | | 0.021  (-0.005,0.047) | | | | | -0.017  (-0.030,-0.003)* | | | | | | | -0.006  (-0.025,0.012) | | | | | -0.029  (-0.047,-0.012)* | | | |  |  |
|  | | | **Lumbar spine, g/cm^2^** | | | | | | | | | | | | | | | | | | | | |  |  |
| Mg | | | -0.00003  (-0.0001,0.00007) | | | | | -0.00008  (-0.0001,-0.00002)* | | | | | | | -0.00003  (-0.0001,0.00006) | | | | | -0.0001  (-0.0002,-0.00003)* | | | |  |  |
| Change very high to high | | | 0.003  (-0.031,0.036) | | | | | 0.010  (-0.010,0.030) | | | | | | | 0.015  (-0.013,0.042) | | | | | 0.003  (-0.022,0.029) | | | |  |  |
| Change from very high to medium | | | -0.008  (-0.044,0.027) | | | | | 0.020  (-0.002,0.042) | | | | | | | 0.015  (-0.014,0.043) | | | | | 0.014  (-0.016,0.044) | | | |  |  |
| Change from very high to low | | | -0.022  (-0.057,0.014) | | | | | 0.010  (-0.010,0.042) | | | | | | | 0.006  (-0.020,0.033) | | | | | 0.021  (0.008,0.049) | | | |  |  |
| Change from very high to very low | | | -0.005  (.0.036,0.025) | | | | | -0.010  (-0.028,0.007) | | | | | | | 0.012  (-0.011,0.034) | | | | | -0.026  (-0.051,-0.002)* | | | |  |  |
| Models adjusted for BMI, leisure-time physical activity (LTPA), diabetes, smoking status, calcium supplements, vitamin D intake, calcium intake, and THR. Nutrient adjusted for energy using the residual method. **p*-value <0.05 | | | | | | | | | | | | | | | | | | | | | | | |  |  |
| **Supplementary Table 6.** Association between selenium intake and BMD. | | | | | | | | | | | | | | | | | | | | | | | |  |  |
|  | | | | **Males (n=327)** | | | | | | **Females (n=991)** | | | | **Females <45 (n=440)** | | | | | | | **Females >=45 (n=551)** | | |  |  |
|  | | | | **β (% 95 CI)** | | | | | | **β (% 95 CI)** | | | | **β (% 95 CI)** | | | | | | | **β (% 95 CI)** | | |  |  |
|  | | | | **Total hip g/cm^2^** | | | | | | | | | | | | | | | | | | | |  |  |
| Se | | | | -0.0002  (-0.0004,0.000001) | | | | | | -0.0003  (-0.0005,-0.0002) * | | | | -0.0002  (-0.0003,-0.000005)* | | | | | | | -0.0004  (-0.0007,-0.0002)* | | |  |  |
| Change very high to high | | | | 0.019  (-0.003,0.040) | | | | | | 0.018  (0.006,0.030) * | | | | 0.013  (-0.005,0.032) | | | | | | | 0.025  (0.007,0.044)* | | |  |  |
| Change from very high to medium | | | | 0.011  (-0.014,0.036) | | | | | | 0.029  (0.017,0.042) * | | | | 0.009  (-0.007,0.025) | | | | | | | 0.037  (0.019,0.056)* | | |  |  |
| Change from very high to low | | | | 0.0004  (-0.022,0.023) | | | | | | 0.001  (0.010,0.012) | | | | 0.0002  (-0.020,0.020) | | | | | | | 0.010  (-0.007,0.026) | | |  |  |
| Change from very high to very low | | | | -0.006  (-0.02,0.012) | | | | | | -0.014(-0.024,-0.004) * | | | | -0.005  (-0.019,0.008) | | | | | | | -0.020  (-0.035,-0.005)* | | |  |  |
|  | | | | **Femoral neck g/cm^2^** | | | | | | | | | | | | | | | | | | | |  |  |
| Se | | | | -0.0003(-0.0005,-0.00001) | | | | | | -0.0003  (-0.0005,-0.0001)* | | | | -0.0001  (-0.0003,0.0001) | | | | | | | -0.0004  (-0.0007,-0.0002)* | | |  |  |
| Change very high to high | | | | 0.024  (-0.005,0.054) | | | | | | 0.017  (0.003,0.030)* | | | | 0.016  (-0.005,0.038) | | | | | | | 0.018  (-0.002,0.038) | | |  |  |
| Change from very high to medium | | | | 0.015  (-0.018,0.049) | | | | | | 0.025  (0.011,0.039)* | | | | 0.002  (-0.017,0.022) | | | | | | | 0.033  (0.013,0.054)* | | |  |  |
| Change from very high to low | | | | 0.011  (-0.020,0.041) | | | | | | -0.004  (-0.016,0.009) | | | | -0.007  (-0.030,0.018) | | | | | | | 0.002  (-0.016,0.020) | | |  |  |
| Change from very high to very low | | | | -0.006  (-0.031,-0.018) | | | | | | -0.019  (-0.030,-0.007)* | | | | -0.007  (-0.023,0.009) | | | | | | | -0.032  (-0.048,-0.015)* | | |  |  |
|  | | | | **Lumbar spine, g/cm^2^** | | | | | | | | | | | | | | | | | | | |  |  |
| Se | | | | 0.0001  (-0.0001,0.0004) | | | | | | -0.0003  (-0.0006,-0.0001)* | | | | -0.0002  (-0.0005,0.0002) | | | | | | | -0.0005  (-0.0009,-0.0002)* | | |  |  |
| Change very high to high | | | | 0.008  (-0.022,0.038) | | | | | | 0.019  (-0.002,0.040) | | | | 0.011  (-0.028,0.050) | | | | | | | 0.011  (-0.017,0.039) | | |  |  |
| Change from very high to medium | | | | 0.007  (-0.028,0.041) | | | | | | 0.028  (0.007,0.049)* | | | | 0.018  (-0.017,0.052) | | | | | | | 0.026  (-0.002,0.055) | | |  |  |
| Change from very high to low | | | | -0.011  (-0.042,0.020) | | | | | | -0.007  (-0.025,0.011) | | | | -0.012  (-0.054,0.030) | | | | | | | -0.014  (-0.039,0.011) | | |  |  |
| Change from very high to very low | | | | -0.006  (-0.031,0.019) | | | | | | -0.017  (-0.034,-0.00001)* | | | | -0.005  (-0.034,0.023) | | | | | | | -0.033  (-0.056,-0.011)* | | |  |  |
| Models adjusted for BMI, leisure-time physical activity (LTPA), diabetes, smoking status, calcium supplements, vitamin D intake, calcium intake, and THR. Nutrient adjusted for energy using the residual method. **p*-value <0.05 | | | | | | | | | | | | | | | | | | | | | | | |  |  |

For instance, decreased consumption of zinc, vitamin E, selenium, and magnesium were associated with lower BMD, but not across all sites. Conversely, decreased vitamin A consumption was associated with higher BMD at the total hip among males. Among women under 45, we found that vitamin A, magnesium, zinc, vitamin E, and selenium intake were associated with lower BMD at the total hip or femoral neck. Most DAI components were significant when the variables were continuous, possibly due to the sample size (**Supplementary Table 1-6**).
